# Editorial: Novel pharmacological approaches targeting mitochondrial dysfunction in diseases

**DOI:** 10.3389/fphar.2022.1041576

**Published:** 2022-10-04

**Authors:** Md. Jamal Uddin, Md. Abdul Hannan, Md. Ataur Rahman, Nadezda Apostolova

**Affiliations:** ^1^ ABEx Bio-Research Center, East Azampur, Dhaka, Bangladesh; ^2^ Graduate School of Pharmaceutical Sciences, College of Pharmacy, Ewha Womans University, Seoul, South Korea; ^3^ Department of Biochemistry and Molecular Biology, Bangladesh Agricultural University, Mymensingh, Bangladesh; ^4^ Department of Pathology, College of Korean Medicine, Kyung Hee University, Hoegidong Dongdaemungu, Seoul, South Korea; ^5^ Korean Medicine-Based Drug Repositioning Cancer Research Center, College of Korean Medicine, Kyung Hee University, Seoul, South Korea; ^6^ Department of Pharmacology, University of Valencia-CIBERehd, Valencia, Spain; ^7^ Foundation for the Promotion of Health and Biomedical Research in the Valencian Region (FISABIO), Valencia, Spain

**Keywords:** mitochondrial dysfunction, oxidative stress, DNA damage, cancers, kidney disease, brain disorders, and novel therapeutics

Because mitochondria play a crucial role in cellular homeostasis and energy balance, mitochondrial dysfunction is critically implicated in the pathobiology of various human diseases/conditions. Redox imbalance, abnormal mitochondrial biogenesis, dysregulated mitophagy, and mitochondrial DNA damage are important causes of mitochondrial dysfunction. Novel pharmacological interventions to improve mitochondrial function hold great promise for the development of future therapies. Research groups from different corners are paying their efforts to address this issue. In this special topic, research and review articles introduce some new insights and strategies which can be capitalized for future development in this field.

While targeting mitochondrial dysfunction in cancer therapy, the use of mitochondria-targeted drugs has some risks, such as abnormal bleeding tendency, since platelet function relies on mitochondrial activity. To address this issue, Montecino-Garrido et al. investigated the impact of three triphenylphosphonium (TPP)-based compounds, including honokiol, lonidamine, and atovaquone on platelet function *in vitro*. They observed that TPP derivatives exhibited an insignificant effect on platelet activation and aggregation, making them a potential antitumor agent with low bleeding risk. However, further studies are imperative to validate whether the findings can be extrapolated to animal models and humans for future clinical use.

Mitochondrial dysfunction is crucially implicated in the pathogenesis of osteoarthritis and regulation of mitochondrial homeostasis through pharmacological intervention may benefit patients with this disease. Provided that mitochonic acid-5 (MA-5) plays an important role in regulating mitochondrial energy metabolism and protecting against mitochondrial damage and activating mitophagy, the potential of MA-5 against IL-1β-induced inflammation in chondrocytes *in vitro* has been explored by Xin et al. They found that MA-5 inhibited IL-1β-induced oxidative stress and protected chondrocytes by upregulating the SIRT3/Parkin-related autophagy pathway, suggesting that MA-5 could be further developed as a potential agent against osteoarthritis.

Moreover, the development of kidney diseases, which has become a public health concern worldwide and is associated with serious clinical complications is also intrinsically linked to mitochondrial dysfunction. Despite the difficulties in drug discovery for kidney diseases, bioactive natural products and their sources have been pursued as a complementary therapeutic approach. Numerous studies have found that small-molecule natural products (SNPs) improve renal function and slow down the course of kidney diseases. In this review, Rahman et al. provide an overview of the nephroprotective properties of SNPs, including berberine, betulinic acid, celastrol, curcumin, polydatin, resveratrol, and salidroside. SNPs have been demonstrated to be effective in treating kidney damage from oxidative stress and mitochondrial DNA damage as well as restoring mitochondrial biogenesis and dynamics in response to a variety of injury stimuli. Thus, such agents need to be acknowledged as multi-target treatments and prospective drugs to slow down the pathogenesis of renal complications, specifically those caused by mitochondrial dysfunction.

Neurodegenerative diseases are a group of neurological conditions that cause structural and functional disorders of the peripheral and the central nervous system which slowly break down over time. The three most frequently diagnosed neurodegenerative disorders are Alzheimer’s disease, Parkinson’s disease, and Huntington’s disease. Unfortunately, the number of people affected by neurodegenerative diseases is growing at an alarming rate. Despite this situation, the drugs currently on the market can rarely interfere with the progression of the disease, in addition to causing significant side effects, which have a heavy impact not only on patients but also on society as a whole. Mounting evidence shows that the emergence of neurodegenerative diseases is directly associated with mitochondrial dysfunction. Apoptosis can take place in nerve cells to varying degrees whenever control of mitochondrial homeostasis breaks down. Li et al. summarize that the natural substances isolated from herbal medicine are effective for the prevention or treatment of neurodegenerative disorders through the management of mitochondrial dysfunction with reduction of other side effects. Therefore, it is important to concentrate on the potential herbal therapeutics that can treat neurodegenerative diseases via inhibiting apoptosis by ameliorating mitochondrial dysfunction. Eventually, this will allow us to lay the groundwork for the development of herbal medicines that can treat neurodegenerative diseases targeting mitochondrial dysfunction.

In summary, we anticipate that this Research Topic will offer detailed settings of mitochondrial dysfunction-related disorders along with recent advances in mitochondria-targeted pharmacological options. Combining these studies will significantly aid in the development of effective therapeutic strategies for treating various diseases associated with mitochondrial dysfunction.

